# The value of microelectrodes and microbipolar electrograms in premature ventricular complex mapping and ablation: The micro-PVC study

**DOI:** 10.1016/j.hroo.2026.03.007

**Published:** 2026-04-12

**Authors:** Marco Bergonti, Nándor Szegedi, Paolo Compagnucci, Olivier Van Leuven, Tardu Özkartal, Maria Luce Caputo, Edit Tanai, Johan Saenen, Michela Casella, Giulio Conte

**Affiliations:** 1 Cardiology department, Cardiocentro Ticino Institute, Ente Ospedaliero Cantonale, Lugano, Switzerland; 2 Cardiology Department, Heart and Vascular Center, Semmelweis University, Budapest, Hungary; 3 Cardiology and Arrhythmology Clinic, University Hospital “Ospedali Riuniti”, Ancona, Italy; 4 Cardiology Department, University Hospital Antwerp, Antwerp, Belgium; 5 Department of Clinical, Special and Dental Sciences, Marche Polytechnic University, Ancona, Italy; 6 Faculty of Biomedical Sciences, Università della Svizzera Italiana, Lugano, Switzerland

**Keywords:** Premature ventricular contractions, Ablation, Ventricular, Microelectrodes, QDOT, Microbipolar

## Abstract

**Background:**

Catheter ablation is an effective treatment for premature ventricular contractions (PVCs). Mapping typically relies on bipolar (bip-EGMs) and unipolar electrograms (uni-EGMs). Recently, catheters equipped with microbipolar electrodes have become available.

**Objective:**

This study aimed to investigate the value of microbipolar EGMs (micro-EGMs) in PVCs mapping.

**Methods:**

We performed a multicenter retrospective study including patients who underwent successful outflow tract PVC ablation using QDOT catheters. 2 groups were identified based on the superficial or intramural origin of the PVC. EGMs—bip-, uni-, and micro-EGMs—were assessed at the successful ablation sites. The primary outcome was the ability of micro-EGM to discriminate between superficial and intramural PVCs. The secondary outcome was signal amplitude.

**Results:**

Among 81 patients (mean age 54 ± 11 years; 59.3% male), 51 had superficial and 30 had intramural PVCs. In superficial PVC, micro-EGM preceded both uni-EGM and bip-EGM in 90% of cases. In particular, when the earliest bip-EGM preceded the earliest micro-EGM by >10 ms (Δ_bip-micro_ >10 ms), there was a high probability (sensitivity 0.94; specificity 0.75) of requiring ablation in both the right ventricular outflow tract and left ventricular outflow tract for successful PVC suppression. Micro-EGMs had higher initial deflection amplitude (1.5 vs 2.8 mV; *P* = .025).

**Conclusion:**

Micro-EGMs allow more accurate PVC mapping, improving depth estimation. Micro-EGMs seem to be valuable for local activation time annotation with important implications on automated annotation algorithms within existing electroanatomic mapping systems.

## Graphical abstract

**Figure:**
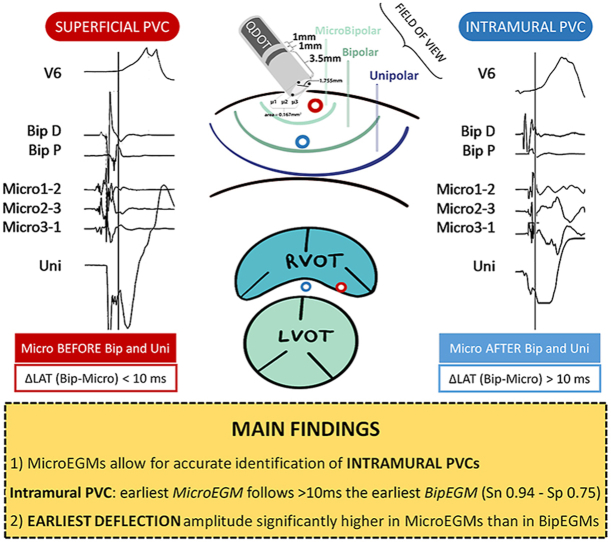

**Key Findings**

▪ Analyzing the relationship among microbipolar, bipolar, and unipolar electrograms (EGMs), it is possible to infer the depth of the premature ventricular contraction (PVC) source.
▪ When the earliest bipolar signal preceded the earliest microbipolar signal by more than 10 ms, there is a high probability (sensitivity of 0.94 and specificity of 0.75) of requiring ablation in both the right and left ventricular outflow tracts for successful PVC suppression.
▪ Microbipolar electrograms (micro-EGMs) provide superior temporal and spatial resolution compared with conventional bipolar and unipolar recordings, enabling more precise localization of PVC origins.
▪ Micro-EGMs seem to be valuable for local activation time annotation with important implications on automated annotation algorithms within existing electroanatomic mapping systems.

## Introduction

Catheter ablation is a well-established therapeutic option for patients with frequent or symptomatic premature ventricular contractions (PVCs), especially for those originating from the outflow tract (OT).^1^ Achieving acute and long-term ablation success heavily depends on accurate activation mapping. In routine practice, both unipolar (uni-EGMs) and bipolar electrograms (bip-EGMs) are used to identify the earliest local activation time (LAT).^2^^,^^3^ The ablation target is often defined by the earliest LAT, typically measured at the downstroke of the unipolar signal, especially when accompanied by a QS pattern, which is considered a marker of proximity to the ectopic focus.^4^^,^^5^ However, this principle is based on 2-dimensional mapping paradigms, which may not be adequate when PVCs arise from regions deeper within the myocardium.^4^^,^^6^

Recently, new ablation catheters with microelectrodes have become available and are used in daily practice.^7^ Microbipolar EGMs (micro-EGMs) have the distinct advantage of capturing near-field tissue activity with minimal interference from far-field structures.^8^ This results in a more localized and precise field of view, effectively excluding distant electrical signals.^9^^,^^10^ Consequently, micro-EGMs have the potential to localize more precisely the source of PVCs, thus improving ablation outcomes. Despite their potential, the value of micro-EGMs in guiding PVC ablation has not been explored.

The objective of our study was to assess the potential of micro-EGMs in distinguishing superficial right ventricular OT (RVOT) PVCs from intramural (OT) PVCs. In particular, we aimed to determine whether (1) the time difference between the earliest micro-EGM vs bip-EGM and uni-EGM can distinguish between superficial and intramural PVC origin, (2) the ablation target area identified by micro-EGM is smaller and more precise than that automatically identified by conventional mapping systems based on uni-EGMs, and (3) micro-EGMs may offer clearer guidance for mapping and ablation than bip-EGMs.

## Methods

### Study design

This was a multicenter retrospective study involving consecutive patients with OT PVCs who underwent successful ablation with the QDOT catheter (Biosense Webster Inc, Irvine, CA). The electroanatomic maps were reviewed offline by expert electrophysiologists, and bip-, micro-, and uni-EGMs at the successful ablation site were collected and analyzed. 2 groups were identified based on the source of the PVC: intramural vs superficial PVC. The EGM characteristics were compared between the 2 groups. Each participating center was responsible for obtaining approval from its local ethics committee, and all patients provided a written informed consent. The study conforms to the Declaration of Helsinki. The datasets generated and/or analyzed during the current study are not publicly available to maintain patient confidentiality but are available from the corresponding author on reasonable request and after the agreement of all the coauthors.

### Patient population

Consecutive patients who underwent OT PVC mapping and ablation with the QDOT catheter between 2022 and 2024 were collected. Only OT PVCs with QRS transition in V3 were enrolled. Patients with unsuccessful PVC ablation or PVC ablated only on the left side (left ventricle [LV] only) were excluded, as were patients in whom the LAT map was incomplete. Comprehensive clinical evaluation was performed in all patients, including medical history, 2-dimensional transthoracic echocardiography, and 12-lead electrocardiograms.

### Definitions and specifics

The QDOT Micro catheter integrates a standard distal bipole (between the 3.5 mm tip and a 1 mm spaced 1 mm ring) recording bip-EGMs with 3 tip microelectrodes (surface area 0.167 mm^2^; 1.755 mm spaced), each recording micro-EGMs at 60° to other micro-EGMs and perpendicular to the standard bip-EGMs. For each patient, micro-, bip-, and uni-EGMs were collected and analyzed at the successful ablation site. A successful ablation site was defined as the ablation site in which the targeted PVC did not recur for >30 minutes after ablation. PVCs effectively eliminated through RVOT-only ablation are considered “superficial” PVCs. PVCs were defined as “intramural” in case ablation from both LVOT and RVOT is needed plus 1 of the following characteristics: (1) equivalently early activation time of <20 ms from adjacent anatomic sites, (2) far-field local bip-EGMs at the sites of earliest activation, (3) absence of QS uni-EGMs at sites of earliest activation, (4) suboptimal pace match (<90%) obtained from sites of earliest activation, and (5) no ventricular arrhythmia suppression or late suppression with early recurrence during radiofrequency (RF) energy delivery on 1 side of the ventricle.^6^^,^^11^, ^12^, ^13^, ^14^, ^15^

### Mapping and ablation procedure

Activation mapping was performed using the ablation catheter with microelectrodes (QDOT, Biosense Webster) and the CARTO system (Biosense Webster). Bip-EGMs (filtered between 30 and 400 Hz) were captured from the end electrode pair of the ablation catheter. Uni-EGMs (filtered from 1 to 240 Hz) were captured from the end electrode of the ablation catheter. Micro-EGMs were collected as bipolar signals between the 3 (A, B, and C) microelectrodes (A–B, B–C, and A–C). An initial map was performed in the RVOT in every patient. If the earliest activation in RVOT was <20 ms before the beginning of the QRS, mapping in the LVOT or the great cardiac vein and the anterior interventricular vein was considered. Ablation locations were chosen based on the initiation of the earliest bip-EGM. Open-irrigation RF power was used, adjusted between 20 and 50 W, with an upper temperature limit of 40°C for durations of 30–90 seconds, aiming for an impedance reduction of 10–15 Ω. If ablation at the primary site in 1 anatomic area did not suppress the PVC, RF was then applied to the earliest activation areas in neighboring structures. A procedure was considered successful if clinical PVC was not observed spontaneously during a waiting period of 20–30 minutes, depending on local practice. All EGMs were acquired with at least 6 g of contact force.

### EGM analysis

Uni-, bip-, and micro-EGMs at the successful ablation site were retrospectively analyzed offline using the CARTO system. Tracings were reviewed by 2 expert electrophysiologists. Measurements were taken using digital calipers within the CARTO system. For each ablation site, LAT was classified as follows: time interval between the earliest deflection in bip-EGM or micro-EGM and QRS onset at any lead (LAT_bip_, LAT_micro_) and time interval between the steepest dV/dT of the uni-EGM and QRS onset at any lead (LAT_uni_), as automatically marked in the CARTO system. A delta(Δ) across LAT_bip_, LAT_micro_, and LAT_uni_ was calculated (Δ_micro-bip_, Δ_micro-uni_, Δ_bip-uni_). EGMs were amplified as needed to accurately assess the earliest deflection on micro-EGM and bip-EGM, even if the earliest deflection did not correlate with a favorable uni-EGM as automated within the mapping system. Near-field signals were annotated.

The presence of QS morphology in the uni-EGM at the ablation site was assessed. The target surface area identified by LAT_uni_ of <20 ms (automatic annotation) and LAT_micro_ of <20 ms was measured as surface area from the CARTO system. The highest amplitude of the first near-field deflection (first 20 ms) for bip-EGM and micro-EGM was collected.

### Focus of the EGM analysis

The primary focus was to assess the ability of micro-EGM to discriminate between superficial and intramural PVCs based on the time difference across LAT_bip_, LAT_micro_, and LAT_uni_. More specifically, the Δ_micro-bip_, Δ_micro-uni_, and Δ_bip-uni_ were compared between superficial and intramural PVCs.

Secondary focuses were (1) a comparison between the target surface area (area of EGM preceding the QRS of >20 ms) identified via an automated algorithm (based on the steepest deflection of uni-EGM) and the 1 identified via micro-EGM and (2) a comparison of the amplitudes of the first deflection (first 20 ms of the EGM) between micro-EGMs and bip-EGMs.

### Clinical follow-up

Patients underwent continuous electrocardiogram Holter monitoring for at least 24 hours to quantify PVC burden at 3 months.

### Statistical analysis and sample size

According to previous literature on >70 patients with OT PVCs, approximately 25% were expected to have intramural PVCs.^6^ This distribution was assessed to have sufficient statistical power to compare the LAT at each successful ablation site for uni-, bip-, and micro-EGMs across the 2 groups and to assess the discriminative power of micro-EGMs LAT compared with conventional measures. Continuous variables were presented as mean ± standard deviation when normally distributed or otherwise as median and interquartile range. Comparisons between groups were undertaken with a parametric (Student *t* test) or nonparametric (Mann–Whitney U) test, respectively. The comparison between categorical variables was performed with the χ^2^ test and Fisher’s exact test, as indicated. A receiver-operating characteristic (ROC) curve was constructed to examine predictive accuracy. The cutoff value was determined based on the ΔLAT value with the highest combined specificity and sensitivity (Youden’s J statistic). A 2-sided *P* < .05 was considered statistically significant for descriptive analysis. Statistical analysis was performed using SPSS 23.0 (IBM Corp, Armonk, NY).

## Results

### Baseline characteristics

**Figure 1:**
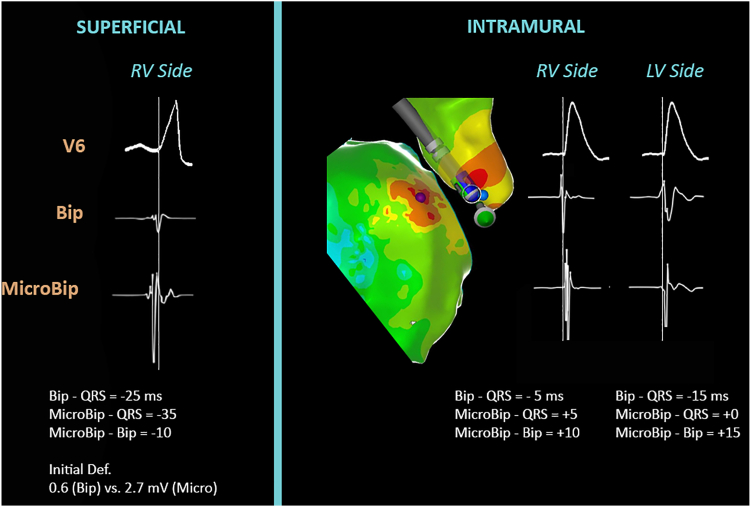
2 representative cases of superficial and intramural premature ventricular contractions. Bip = bipolar; LV = left ventricle; MicroBip = microbipolar; RV = right ventricle.

A total of 81 patients were included, of whom 51 were in the superficial PVC group (mean age 52.0 years) and 30 (37%) in the intramural group (mean age 59.3 years). 2 representative cases are presented in Figure 1. Mean PVC burden was 24.1% ± 11.4%, and preablation LV ejection fraction was <50% in 28.4%, in the absence of known cardiomyopathy (ie, suspected PVC-induced cardiomyopathy). Patients in the intramural PVC group were more often male (43.1% vs 70.0%). The remaining baseline characteristics were not significantly different between the 2 groups and are presented in Table 1.

**Table 1:** Characteristics of the participants at baseline

Characteristic	Superficial PVC (n = 51)	Intramural PVC (n = 30)	*P* value
Age, y, mean ± SD	52.0 ± 18.7	59.3 ± 14.6	.071
Male sex, n (%)	22 (43.1)	21 (70.0)	.035
PVC burden, %	23.0 ± 15.0	26.9 ± 8.4	.21
Previous PVC ablation, n (%)	7 (13.8)	5 (16.7)	.97
LVEF, %	55.6 ± 9.7	51.4 ± 11.6	.092
LVEF <50%, n (%)	11 (21.6)	12 (40.0)	.13

LVEF = left ventricular ejection fraction; PVC = premature ventricular contraction; SD = standard deviation.

### Intraprocedural data and follow-up

Intraprocedural data are presented in Table 2. Absolute intraobserver variability of 10 randomly selected cases measured at least twice with the CARTO systems was 1.5 ± 1.1 ms. Among patients in the superficial PVC group, the posteroseptal RVOT was identified as the origin of the PVC in the majority of the cases (61.2%). Among patients in the intramural PVC group, the earliest activation was recorded on the RVOT in 47.5% and on the LVOT in the remaining 52.5%. In the superficial PVC group, the earliest mean LAT_bip_ was 30.2 ± 10.6 ms (difference between the earliest bip-EGM and the beginning of QRS), whereas the earliest LAT_micro_ was 31.6 ± 12.6 ms. In the intramural PVC group, the earliest mean LAT_bip_ on the right and left sides was, respectively, 21.6 ± 6.4 ms and 19.1 ± 10.0 ms. The earliest LAT_micro_ on the right and left sides was, respectively, 9.7 ± 12.4 and 6.9 ± 5.9 ms. A unipolar QS morphology was reported in 76.5% of the successful ablation sites in the superficial PVC group and in 36.7% of the intramural PVC. Ablation time was significantly longer on the intramural PVC group (183.6 vs 397.8 seconds; *P* = .001). There were no procedure-related major complications. All patients had long-term PVC suppression (burden reduction >90%). The mean distance between neighboring points in the LV and right ventricular regions in patients with intramural PVC was 1.2 cm (VisiTag to VisiTag) ± 0.6 cm.

**Table 2:** Intraprocedural data

Characteristic	Superficial PVC (n = 51)	Intramural PVC (n = 30)	*P* value
Earliest activation site, n (%)			
Posteroseptal RVOT	31 (60.8)	8 (26.7)	.005
Anteroseptal RVOT	17 (33.3)	6 (20.0)	.06
RVOT free wall	3 (5.9)	-	1.00
LVOT RCC	-	3 (10.0)	-
LVOT LCC	-	5 (16.7)	-
RCC/LCC junction	-	8 (26.7)	-
Earliest LAT compared with QRS onset, ms, mean ± SD			
RVOT LAT_bip_	30.2 ± 10.6	21.6 ± 6.4	<.001
RVOT LAT_uni_	27.9 ± 9.8	12.6 ± 11.3	<.001
RVOT LAT_micro_	31.6 ± 12.6	9.7 ± 12.4	<.001
LVOT LAT_bip_		19.1 ± 10.0	-
LVOT LAT_uni_		13.8 ± 11.7	-
LVOT LAT_micro_		6.9 ± 5.9	-
Difference between LATs, ms, mean ± SD			
RVOT Δ_micro-bip_	0 (0–5.0)	−17.0 (−19.0 to −3.8)	<.001
RVOT Δ_uni-bip_	0 (−4.0 to 0)	−14.0 (−15.0 to −1.0)	<.001
RVOT Δ_micro-uni_	0 (0–10)	−4.0 (−5.0 to −0.8)	<.001
LVOT Δ_micro-bip_		−11.1 (−22.0 to −1.5)	-
LVOT Δ_uni-bip_		−3.8 (−10.6 to 0)	-
LVOT Δ_micro-uni_		−4.4 (−15.5 to 0.3)	-
QS on unipolar RVOT, n (%)	39 (76.5)	11 (36.7)	<.001
QS on unipolar LVOT, n (%)	-	19 (63.3)	-
Ablation time, s	183.6 ± 95.5	397.8 ± 134.4	.001

bip = bipolar; LAT = local activation time; LCC = left coronary cusp; LVOT = left ventricular outflow tract; micro = microbipolar; PVC = premature ventricular contraction; RCC = right coronary cusp; RVOT = right ventricular outflow tract; SD = standard deviation; uni = unipolar.

### Differences in Δ LAT between superficial and intramural PVCs

**Figure 2:**
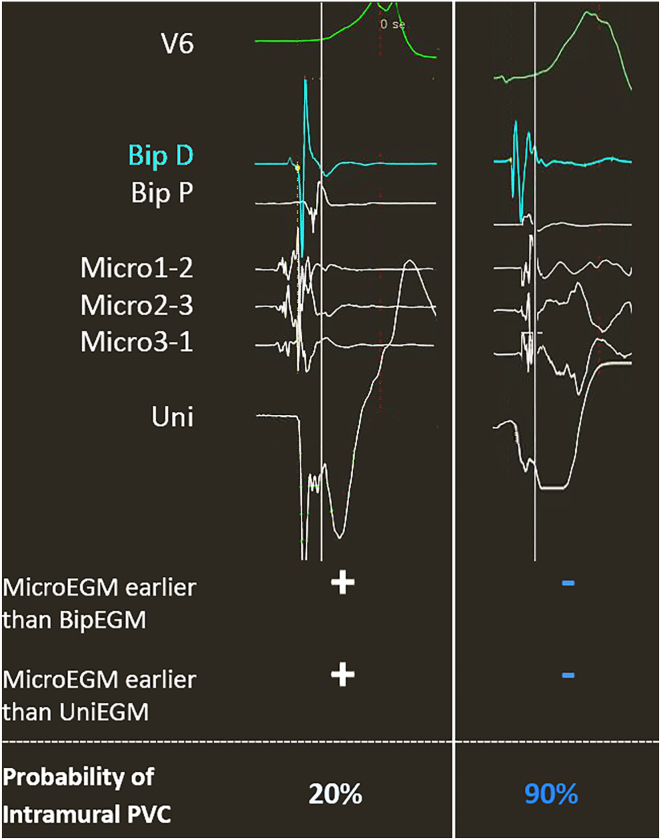
Examples of superficial and intramural PVCs, characterized by the time relationship among micro-, bip-, and uni-EGMs. bip = bipolar; EGM = electrogram; micro = microbipolar; PVC = premature ventricular contraction; uni = unipolar.

**Figure 3:**
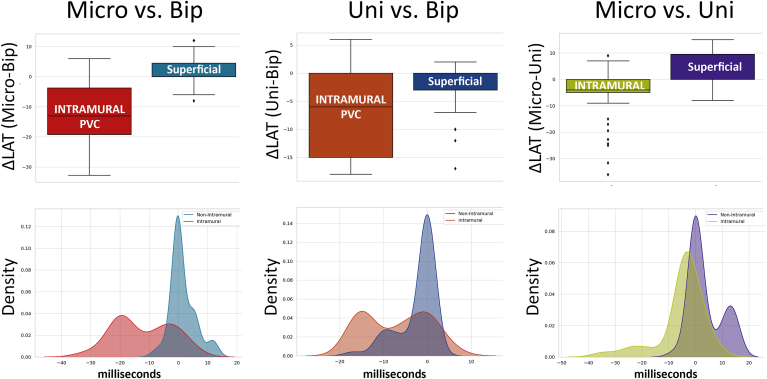
Distribution of ΔLAT of micro-, bip-, and uni-EGMs in superficial and intramural PVCs. bip = bipolar; EGM = electrogram; LAT = local activation time; micro = microbipolar; PVC = premature ventricular contraction; uni = unipolar.

When the earliest micro-EGM preceded both the earliest bip-EGM and the earliest uni-EGM, the probability of intramural PVC was 20%. When the micro-EGM was later than the bip-EGM and the uni-EGM, the probability of intramural PVC was 90% (Figure 2). Δ_micro-bip_ was significantly smaller in the superficial PVC group than the intramural PVC group: 0 ms (0–5.0) vs 17 ms (19.0–3.8) on the RVOT side and 11.1 ms (22.0–1.5) on the LVOT side (Table 2 and Figure 3). Δ_micro-uni_ was also significantly smaller in the superficial PVC group than the intramural PVC group: 0 ms (0–10.0) vs 4.0 ms (0.8–5.0) on the RVOT side and 4.4 ms (15.5–0.3) on the LVOT side (central illustration).

### Cutoff values and prediction of intramural PVC

**Figure 4:**
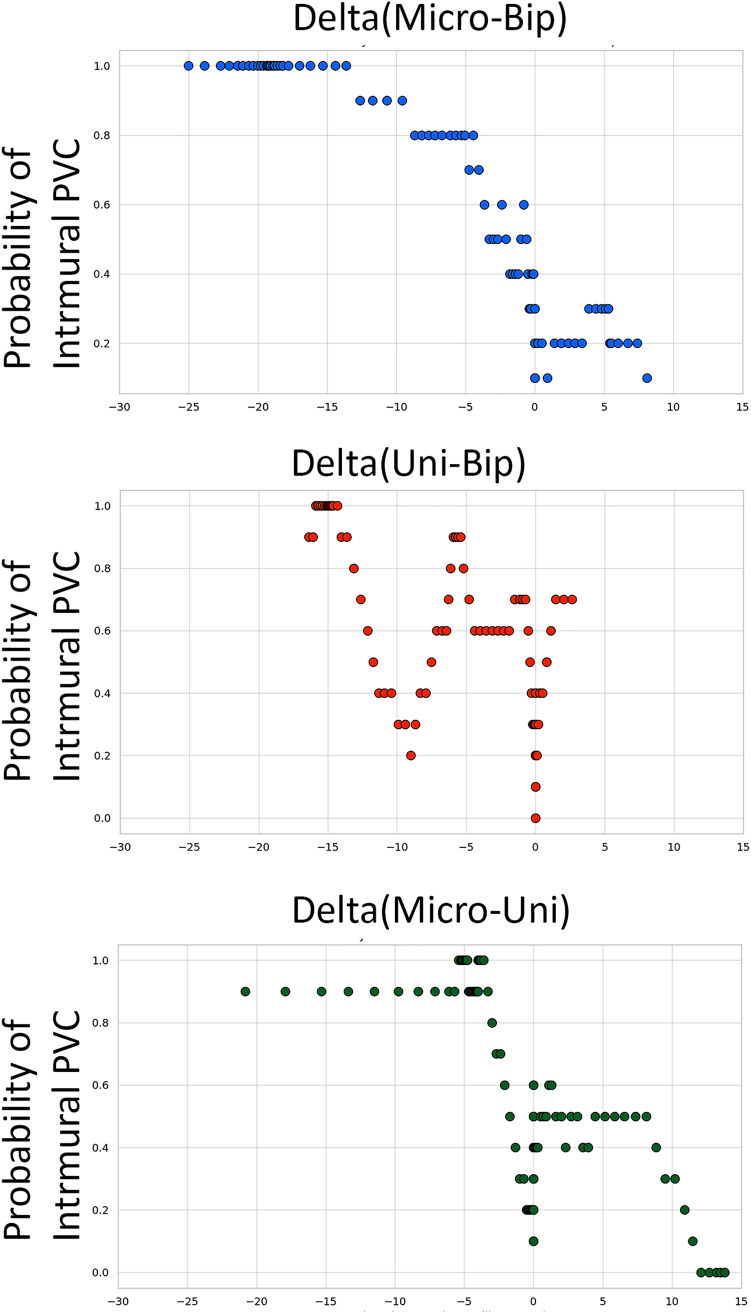
Relationship of overall Δlocal activation time and the probability of requiring right ventricular outflow tract + left ventricular outflow tract ablation. bip = bipolar; micro = microbipolar; PVC = premature ventricular contraction; uni = unipolar.

Comparing the ROC curves of Δ_bip-micro_, Δ_bip-uni_, and Δ_micro-uni_, the first one was the one showing better accuracy (Table 3) with an area under the curve of 0.89 (0.81–0.95; *P* < .001). The best cutoff value was found to be −10 ms (micro-EGM following the bip-EGM by >10 ms), with a sensitivity of 0.94 and a specificity of 0.75 for identifying intramural PVC. The relationship of each ΔLAT and the probability of intramural PVC is presented in Figure 4.

**Table 3:** AUC

Delta	AUC	Best cutoff	Sensitivity	Specificity	*P* value
Δ_bip-micro_	0.89 (0.81–0.95)	−10.0 ms	0.94	0.75	<.001
Δ_bip-uni_	0.70 (0.6–0.80)	−6.5 ms	0.71	0.72	.05
Δ_uni-micro_	0.82 (0.73–0.89)	−8.5 ms	0.96	0.65	.04

AUC = area under the curve; bip = bipolar; micro = microbipolar; uni = unipolar.

### Target area and amplitude of the first deflection

Among patients with superficial PVC, the target ablation area (area of EGMs earlier than 20 ms than QRS) was significantly smaller when the reference EGM was the micro-EGM than the automatic annotation performed on the steepest uni-EGM: 1.5 cm^2^ vs 3.0 cm^2^ (*P* = .016). The amplitude of the earliest deflection was significantly higher in the micro-EGMs than the bip-EGMs (1.5 vs 2.8 mV; *P* = .025).

## Discussion

This is, to our knowledge, the first study to investigate the role of microelectrodes and micro-EGMs in improving mapping and ablation of OT PVCs. The 3 main findings are as follows: (1) Microelectrodes allow accurate identification of intramural PVCs; as in these cases, the earliest micro-EGM is significantly later than bip-EGMs and uni-EGMs. In particular, when the earliest micro-EGM follows by >10 ms the earliest bip-EGM, there is a high probability (sensitivity 0.94; specificity 0.75) of requiring ablation in both the RVOT and LVOT for successful PVC suppression. Later micro-EGM in intramural foci reflects a narrower field of view capturing true endocardial activation, whereas earlier bipolar/unipolar components may include far-field from intramural sources. (2) The amplitude of the earliest deflection (first 20 ms) is 2-fold higher in micro-EGMs than bip-EGMs, allowing for easier and more accurate annotation. (3) Manual annotation on micro-EGMs reduces the target ablation area (defined as EGMs occurring >20 ms before QRS onset) by approximately 50% compared with automatic annotation.

### Microelectrode properties and differences compared with bipolar and unipolar electrodes

The rationale supporting the study hypothesis is rooted in the distinct physical characteristics of unipolar and bipolar signals. Unipolar recordings possess a broader field of view and are less sensitive to detecting low-amplitude, near-field EGMs.^10^ In contrast, bipolar signals—obtained by subtracting 2 unipolar recordings—better highlight these local, low-amplitude signals.^10^ The characteristics of a bip-EGM are influenced by factors such as electrode size, the orientation of the bipole relative to the wavefront, and the angle formed between the catheter and the tissue. Smaller electrodes positioned closely together tend to produce high-frequency, sharper signals with shorter durations.^9^^,^^10^ In this context, the QDOT catheter represents a meaningful advancement in the mapping of focal arrhythmias. It features 3 microelectrodes arranged in a triangular configuration, with each bipole separated by 60°. This design offers several key benefits. Compared with conventional electrodes, microelectrodes can record signals with amplitudes up to 3 times greater at the same location.^8^^,^^9^^,^^16^ This increased signal amplitude may facilitate easier and more accurate EGM annotation.^8^^,^^9^^,^^16^ Moreover, their smaller field of view enhances the ability to isolate local signals by minimizing far-field interference. The 3 bipoles are oriented 60° apart, enabling signal detection from multiple directions. This multidirectional capability allows for recordings regardless of the orientation of the incoming wavefront.

Although the benefits of microelectrode and micro-EGM recordings have been hypothesized and demonstrated in preclinical models, including in vitro settings and animal studies, their impact has not been evaluated in clinical practice. Our study is the first to demonstrate the added value of microelectrodes and micro-EGMs in patients who underwent PVC ablation. Our findings confirm the superior signal clarity provided by microelectrodes, with recorded signals displaying amplitudes up to twice as high as those obtained with conventional bipolar recordings. This improved signal quality facilitates easier and more confident annotation of LATs.^17^ Beyond amplitude, our data also support the concept that microelectrodes provide enhanced spatial resolution and reduced far-field interference. This is evidenced by the fact that the target region identified using micro-EGM–based annotation was approximately 50% smaller than the area delineated automatically by the standard mapping system. Importantly, in cases of intramural PVCs, a consistent delay was observed in the LAT recorded with micro-EGMs compared with conventional bip-EGMs. This temporal offset reinforces the notion that microelectrodes, owing to their narrower field of view, capture only the true endocardial activation, which occurs later in these cases than the earlier far-field signals detected by both bipolar and unipolar recordings that reflect deeper intramyocardial activity.^18^^,^^19^ The observation that 10% of superficial PVCs showed a later micro-EGM than the bip-EGM likely reflects temporal simultaneity rather than true signal delay, whereas in 20% of intramural PVCs the earlier micro-EGM may be explained by its greater signal amplitude allowing annotation of activity not visible on the bip-EGM and by differential effects of catheter–tissue angulation on bip-EGM vs micro-EGM recordings. Overall, these findings highlight the potential of catheters equipped with microelectrodes to guide and possibly enhance the precision and efficacy of PVC mapping and ablation. Further studies are needed to determine whether small-tip catheters equipped with microelectrodes or multipolar catheters incorporating multipolar or omnipolar technologies offer the greatest advantage for guiding PVC ablation.^20^, ^21^, ^22^ In addition, with the advent of pulsed field ablation, the ability to distinguish between superficial and intramural PVCs may help guide the use of more aggressive ablation protocols, incorporating both RF ablation and pulsed field ablation, to achieve deeper lesions when the target is intramural.

### Comparison with previous studies

This is the first study that systematically compares micro-EGM with uni-EGM and bip-EGM in successfully ablated OT PVC.

The utility of uni-EGM morphologies—particularly QS—for guiding ablation of focal arrhythmias comes from accessory pathways ablation.^23^ Their relatively superficial location simulating a 2-dimensional geometry makes these arrhythmias suitable for unipolar signal interpretation. In subsequent studies analyzing QS morphology in PVC mapping, it was shown that QS morphologies could be observed even 11 mm away from the pacing source, suggesting poor spatial specificity.^24^ For this reason, interest slowly shifted toward bip-EGM. In a more recent study, 66 patients with various PVC origins (including OT and LV summit) were analyzed and arrhythmia depth was classified based on RF response time. It was noted that although the earliest sharp bip-EGM correlated with effective ablation, the steepest uni-EGM dV/dT often occurred later, particularly in intramural cases.^4^ The delay between bip-EGM and uni-EGM (Δ_bip-uni_) was significantly lower in superficial PVCs (3.5 ms) than in intramural ones (25.5 ms) or failed ablations (12.5 ms). These findings were recently confirmed in a medium-sized retrospective analysis of 70 patients who underwent successful OT PVC ablation.^6^ This study showed that the earliest bip-EGM activation guides successful ablation of OT PVCs better than uni-EGM–guided analysis, especially when an intramural PVC source is present. In addition, the authors were able to show that Δ_bip-uni_ of >15 ms best distinguished sites in which RVOT-only vs RVOT + LVOT ablation achieved acute PVC suppression.

Our findings are consistent with and further support existing literature comparing bip-EGMs and uni-EGMs. As illustrated in Table 2, in the context of intramural PVCs, the bip-EGM preceded the uni-EGM by approximately 10 ms, and a QS morphology at the successful ablation site was observed in only 37% of these cases. This low incidence of QS patterns in intramural PVCs reinforces previous observations regarding the limited reliability of uni-EGM morphology in such settings. Furthermore, the ROC curve of the Δ_bip-uni_ in our study was 0.7, closely matching the previously reported value of 0.75.^6^ Importantly, our study moves beyond these established findings by introducing new insights into the role of micro-EGMs. In particular, ROC curve analysis revealed superior diagnostic performance for micro-EGMs compared with the conventional bipolar-unipolar, with an area under the curve of 0.89 vs 0.70. This result highlights the added value of micro-EGMs in distinguishing intramural PVCs. Taken together, our data support the use of the earliest deflection on the micro-EGM and the temporal difference between micro-EGM and bip-EGM signals as novel and promising criteria to accurately localize intramural PVC origins and guide effective ablation. This result aligns with previous studies on micro-EGMs applied in the setting of sustained ventricular arrhythmia ablation, showing that micro-EGMs are more sensitive than standard bipolar in the identification of viable myocytes in sinus rhythm and may facilitate recognition of the critical isthmus during ventricular tachycardia.^8^

### Limitations

This study was a retrospective analysis, and as such, its interpretation must be approached with caution owing to inherent limitations. The identification of the site of successful ablation may be imprecise in cases where multiple lesions were necessary to achieve PVC suppression, particularly in the intramural PVC group. Although multiple operators contributed to individual procedures and retained some discretion in the mapping strategy, a standardized stepwise approach—beginning with mapping and ablation of earliest RVOT sites and progressing to adjacent anatomic regions—is routinely applied at our institutions. Although the inclusion of PVCs from clearly intramural sites may have further strengthened the analysis, we intentionally selected cases in which uniform procedural success could be achieved using standard ablation strategies and a limited number of lesions. In addition, our analysis was restricted to patients mapped and ablated using the CARTO system and the QDOT catheter. Further studies are warranted to prove the same finding in other catheters embedded with microelectrodes. Catheter orientation influences bip-EGM acquisition, and this may introduce a bias in the EGM interpretation in a specific heart location. Intracardiac echocardiography was not used in this study, which may have limited the precision in defining valvular landmarks such as the pulmonary valve level and aortic cusps and, consequently, the exact anatomic localization of catheter position during mapping and ablation. A direct comparison between bip-EGM configurations was not performed, given that bipolar signals were analyzed using a standardized filter setting (30–400 Hz) based on previous literature; therefore, differences in diagnostic performance between bip-EGM interpretation and micro-EGM analysis could not be systematically assessed.^25^ Finally, only intramural sites at the OT were evaluated. Whether this applies to other intramural regions should be evaluated.^26^

## Conclusion

Our study demonstrates that microelectrodes and micro-EGMs may improve our understanding and mapping of OT PVCs by enabling the identification of intramural origin. In particular, when the earliest micro-EGM follows the earliest bip-EGM by >10 ms, this predicts the need for combined RVOT and LVOT ablation. In addition, micro-EGMs enhance signal clarity and enable a 50% reduction in ablation area compared with automatic annotation based on uni-EGMs. Micro-EGMs seem to be valuable for LAT annotation with important implications on the automated annotation algorithms within existing electroanatomic mapping systems.

## Disclosures

M.B. reports receiving speaker honoraria from Johnson & Johnson, consulting fees from Boston Scientific, and a research grant from Johnson & Johnson and Boston Scientific. N.S. reports consulting fees from Boston Scientific, Abbott, and Johnson & Johnson. The authors declare that all illustrations and figures in the manuscript are entirely original and do not require reprint permission. The authors have no conflicts of interest to disclose.
